# The Use of Artificial Intelligence and Wearable Inertial Measurement Units in Medicine: Systematic Review

**DOI:** 10.2196/60521

**Published:** 2025-01-29

**Authors:** Ricardo Smits Serena, Florian Hinterwimmer, Rainer Burgkart, Rudiger von Eisenhart-Rothe, Daniel Rueckert

**Affiliations:** 1 Department of Orthopaedics and Sports Orthopaedics Klinikum rechts der Isar Technical University of Munich Munich Germany; 2 Institute for AI and Informatics in Medicine Technical University of Munich Munich Germany

**Keywords:** artificial intelligence, accelerometer, gyroscope, IMUs, time series data, wearable, systematic review, patient care, machine learning, data collection

## Abstract

**Background:**

Artificial intelligence (AI) has already revolutionized the analysis of image, text, and tabular data, bringing significant advances across many medical sectors. Now, by combining with wearable inertial measurement units (IMUs), AI could transform health care again by opening new opportunities in patient care and medical research.

**Objective:**

This systematic review aims to evaluate the integration of AI models with wearable IMUs in health care, identifying current applications, challenges, and future opportunities. The focus will be on the types of models used, the characteristics of the datasets, and the potential for expanding and enhancing the use of this technology to improve patient care and advance medical research.

**Methods:**

This study examines this synergy of AI models and IMU data by using a systematic methodology, following PRISMA (Preferred Reporting Items for Systematic Reviews and Meta-Analyses) guidelines, to explore 3 core questions: (1) Which medical fields are most actively researching AI and IMU data? (2) Which models are being used in the analysis of IMU data within these medical fields? (3) What are the characteristics of the datasets used for in this fields?

**Results:**

The median dataset size is of 50 participants, which poses significant limitations for AI models given their dependency on large datasets for effective training and generalization. Furthermore, our analysis reveals the current dominance of machine learning models in 76% on the surveyed studies, suggesting a preference for traditional models like linear regression, support vector machine, and random forest, but also indicating significant growth potential for deep learning models in this area. Impressively, 93% of the studies used supervised learning, revealing an underuse of unsupervised learning, and indicating an important area for future exploration on discovering hidden patterns and insights without predefined labels or outcomes. In addition, there was a preference for conducting studies in clinical settings (77%), rather than in real-life scenarios, a choice that, along with the underapplication of the full potential of wearable IMUs, is recognized as a limitation in terms of practical applicability. Furthermore, the focus of 65% of the studies on neurological issues suggests an opportunity to broaden research scope to other clinical areas such as musculoskeletal applications, where AI could have significant impacts.

**Conclusions:**

In conclusion, the review calls for a collaborative effort to address the highlighted challenges, including improvements in data collection, increasing dataset sizes, a move that inherently pushes the field toward the adoption of more complex deep learning models, and the expansion of the application of AI models on IMU data methodologies across various medical fields. This approach aims to enhance the reliability, generalizability, and clinical applicability of research findings, ultimately improving patient outcomes and advancing medical research.

## Introduction

The integration of advanced computational methods such as artificial intelligence (AI) in medicine represents a significant leap in the pursuit of more accurate, efficient, and personalized health care [1]. The increasing popularity of wearable devices has led to a surge in the collection of various physiological signals, including accelerometer data from wristbands, smartwatches, and other sensors [2]. While these wearables offer valuable insights into our daily activities, inertial measurement units (IMUs) stand out for their unique ability to capture 3-dimensional motion data, including acceleration, angular velocity, and orientation. Furthermore, IMUs are also highly accessible and widely available, making them an attractive choice for researchers and clinicians alike. This combination of precision and accessibility has led to a surge in the adoption of IMUs across various medical fields, from neurology to emergency medicine, where they are increasingly being used to track complex motor behaviors, such as those seen in neurological disorders or injuries.

The ability of AI models to extract meaningful patterns from complex, multidimensional movement data offers unprecedented opportunities in diagnostics, patient monitoring, and treatment efficacy assessment. However, despite the potential benefits, there are many challenges in applying these technologies in a medical context [1]. These include issues related to data preprocessing, model selection, dataset size, and study setting (dataset bias), which can significantly impact the validity and reliability of the results. In addition, integrating these new technologies into existing clinical workflows poses significant logistical and ethical challenges, including data privacy concerns, the need for extensive validation, and the training of health care professionals to effectively use these tools.

This systematic review aims to provide a comprehensive overview of the current state of AI applications in processing IMU data for medical purposes. By examining a variety of studies across different medical specialties, this review seeks to understand the common methodologies, tools, and challenges faced in this rapidly evolving field. In doing so, it aims to identify gaps in the current knowledge base, suggest areas for future research, and provide insights into the practical implications of these technologies in clinical settings. Ultimately, the goal is to foster a deeper understanding of how AI algorithms and IMUs can be harnessed to improve patient outcomes and streamline health care delivery.

## Methods

### Search Strategy

The design of this systematic review was centered around 3 fundamental research questions, all pertaining to the application of AI models in analyzing IMU data within various medical fields. The first question aimed to identify which medical fields are most actively incorporating such techniques with IMU data. The second question focused on determining the specific models that are used in the analysis of IMU data. Finally, the third question tries to understand the characteristics of the datasets used for training. To address these questions, this review follows the PRISMA (Preferred Reporting Items for Systematic Reviews and Meta-Analyses) guidelines [3] to ensure a methodical approach to data collection and analysis, with the PRISMA checklist provided in Multimedia Appendix 1.

The literature search was conducted on November 15, 2023, using 2 major scientific databases: PubMed and Web of Science. PubMed yielded 327 articles with the search string: (“Artificial Intelligence”[Mesh]) AND (health OR medical OR clinical OR patient) AND (wearabl* OR smartphone OR smartwatch) AND (magnetometer OR accelerom* OR gyroscope OR “imu” OR “imus” OR “Inertial measurement unit”) AND (health OR medical OR clinical OR patient) NOT (Review[Publication Type] OR Systematic Review[Publication Type]).

Web of Science yielded 853 articles with the search string: (“Artificial Intelligence” OR “Machine Learning” OR “Deep Learning”) AND (health OR medical OR clinical OR patient) AND (wearabl* OR smartphone OR smartwatch) AND (magnetometer OR accelerom* OR gyroscope OR “imu” OR “imus” OR “Inertial measurement unit”) AND (health OR medical OR clinical OR patient).

### Study Selection

The retrieved articles were screened by 2 Computer Scientists (RSS and FH) based on their titles and abstracts to assess their relevance to the research questions (inclusion and exclusion criteria outlined in Textbox 1).

**Guidelines for selecting studies from the literature research.**

**Inclusion criteria**

- Study uses 1 or more inertial measurement units.
- Study targets a clinical condition.
- Study focuses on machine learning or deep learning algorithms for data analysis.
- Study published in English.
- Studies with available full-text articles.
- Studies with more than 20 participants.

**Exclusion criteria**

- Review articles.
- Studies not related to medical applications.
- Studies not using inertial measurement unit data.
- Study not mentioning details on the machine learning or deep learning techniques used.

### Data Extraction

In each included study, relevant data were extracted, concentrating on several key aspects. This included the medical field of application, the specific purpose of using machine learning/deep learning (ML/DL), and the ML/DL methods. In addition, the size of the study dataset and the study setting were used. The synthesized data from this extraction process provided a comprehensive overview, highlighting the current state of ML/DL applications in processing IMU data for medical purposes. Based on research suggesting that participant diversity is required for good generalization [4], we consider dataset size to be the number of participants.

## Results

### Overview

**Figure 1:**
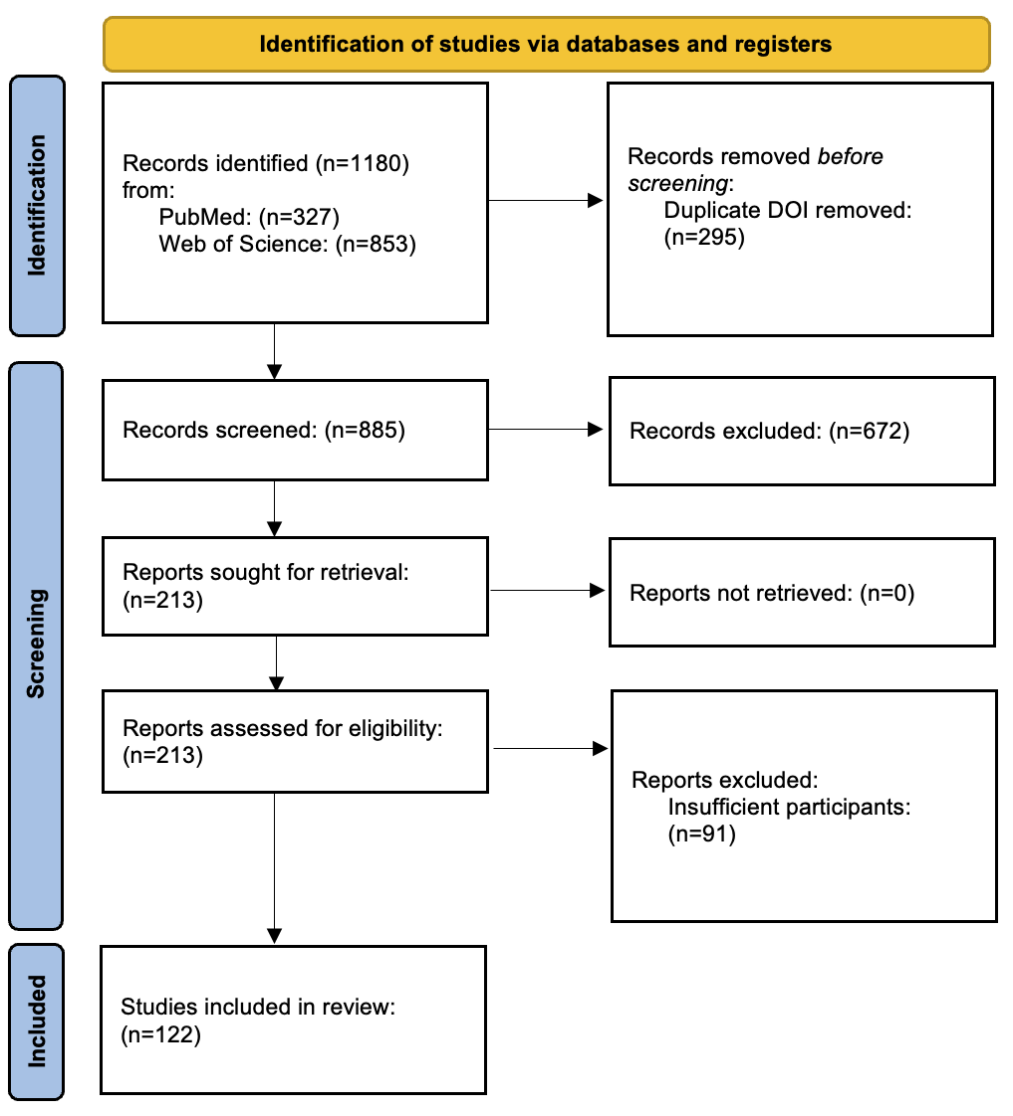
PRISMA (Preferred Reporting Items for Systematic Reviews and Meta-Analyses) flow diagram of the filtering procedure applied to bibliographic search results.

**Figure 2:**
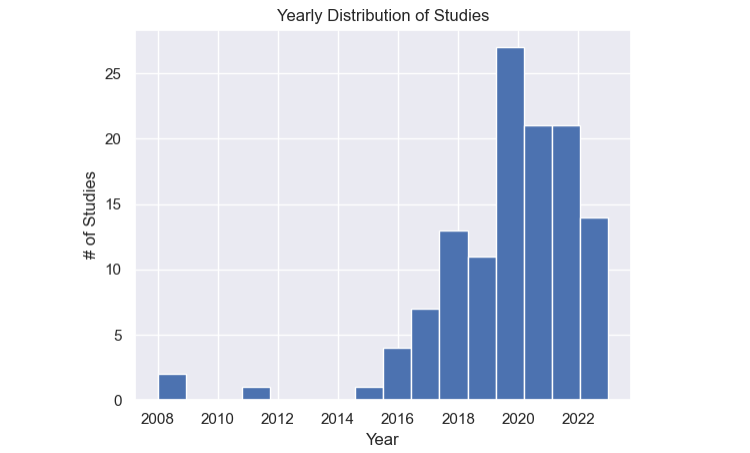
Histogram of annual publication frequency of the final 122 studies.

This systematic review initially identified 1180 papers through a comprehensive search. After the screening process, 122 papers from the years 2008 to 2023 were selected for detailed analysis. The PRISMA diagram illustrating this selection process is presented in Figure 1, and the distribution of these papers over the years is found in Figure 2. The results are organised based on predefined research questions, providing a detailed overview of the current landscape, prevalent trends, and challenges in this domain.

### Distribution of Papers

Neurology stood out as the most represented field, with a substantial count of 79 (64.7%) papers, underscoring its significant role in the application of IMU data. Geriatric medicine followed, with 12 (9.8%) papers, reflecting an interest in this area for elderly care. Pulmonary and cardiology medicine, with 10 (8.1%) papers, indicated their importance in chronic disease management using these technologies. Musculoskeletal and orthopedic care was another notable field, represented by 8 (6.5%) papers, emphasizing the role of AI in diagnosing and treating musculoskeletal disorders. Pediatric medicine and psychiatry also featured in the analysis, with 4 (3.2%) and 2 (1.6%) papers respectively, highlighting some areas of opportunity. In addition, fields such as Emergency medicine, general and preventive medicine, internal medicine, oncology, otolaryngology, sleep medicine, and vestibular medicine each appearing one time, in total 7, demonstrating the wide-ranging applications of IMU data across various medical disciplines.

In analyzing the applications of AI models on medical IMU data, disease monitoring emerged as the primary focus, with 38 (31.1%) papers dedicated to this aspect. This indicates a strong trend toward using ML models for ongoing patient care and management. Disease detection and disease severity assessment were also prominent, with 19 (15.5%) and 24 (19.6%) papers respectively, highlighting the interest early diagnosis and evaluation of disease progression. Functional and mobility assessment, covered in 13 (10.6%) papers, pointed to the importance of AI in evaluating patients’ physical abilities. Rehabilitation and recovery monitoring, with 10 (8.1%) papers, further emphasized the use of AI in patient recovery processes. Risk prediction and preventive analysis, represented by 18 (14.7%) papers, showed a proactive approach in using AI for forecasting health risks and preventing diseases (Table 1).

**Table 1:** List of studies’ references ordered by medical field and machine learning purpose.

Medical field and AI^a^ purpose		Studies	Total, n
**Neurology**			
	Disease monitoring	[5-32]	28
	Disease severity assessment	[33-50]	18
	Disease detection	[51-62]	12
	Functional and mobility assessment	[63-70]	8
	Risk prediction and preventive analysis	[71-77]	7
	Rehabilitation and recovery monitoring	[78-83]	6
**Geriatrics**			
	Risk prediction and preventive analysis	[84-91]	8
	Functional and mobility assessment	[92,93]	2
	Disease monitoring	[94]	1
	Disease severity assessment	[95]	1
**Cardiorespiratory**			
	Disease detection	[96-101]	6
	Disease monitoring	[102-105]	4
	Rehabilitation and recovery monitoring	[106]	1
**Orthopedics**			
	Disease monitoring	[107,108]	2
	Disease severity assessment	[53,109]	2
	Rehabilitation and recovery monitoring	[110,111]	2
	Functional and mobility assessment	[112]	1
	Risk prediction and preventive analysis	[113]	1
**Pediatrics**			
	Functional and mobility assessment	[114,115]	2
	Disease monitoring	[116]	1
	Disease severity assessment	[117]	1
**Psychiatry**			
	Disease monitoring	[118,119]	2
**Emergency Medicine**			
	Risk prediction and preventive analysis	[120]	1
**Preventive medicine**			
	Rehabilitation and recovery monitoring	[121]	1
**Oncology**			
	Risk prediction and preventive analysis	[122]	1
**Otolaryngology**			
	Disease detection	[123]	1
**Sleep medicine**			
	Disease severity assessment	[124]	1
**Vestibular medicine**			
	Disease severity assessment	[125]	1

### Machine Learning and Deep Learning Models Used in Inertial Measurement Unit Data Analysis

In total, 326 models were identified across the 122 reviewed papers, averaging 2.67 tested models per paper. The analysis of the AI models used for analyzing IMU data revealed distinct trends in the usage of both ML and DL approaches. The results are presented in 2 subsections detailing the prevalence and variety of models observed in the reviewed papers. Performance metrics were not included in the analysis because studies often used different metrics, making direct comparison impossible. Table 2 provides a list of all studies sorted by the AI model used. If a study employed more than one model, it appears multiple times. A full description of the model names is provided in Multimedia Appendix 2.

**Table 2:** List of studies sorted by artificial intelligence model used in the study.

Name	Studies	Total, n
Support vector machine	[6,7,9-13,15,17,20,21,25-27,31,33,36,38-43,45-48,52,56-58,62,65,66,68,72-75,81,87,88,93,94,96,98, 101,104-106,110,111,113,115,119,121,123,125,126]	61
Random forest	[7,9,11,16,17,25,29,38-40,44,45,47,48,51,55,56,58,61,65-68,73,74,77,80,82,87,91,94,95,97,98,104,105,111,114, 115,117,121,123,126]	43
k-nearest neighbors	[10,20,22,27,33,36,40,41,43,44,47,52,56,58,59,62,65,73,81,87,91,95,111,117,123]	25
Convolutional neural network	[7-9,17,18,23,29,30,39,53,54,63,64,78,85,94,103,107,120,124]	20
Linear regression	[5,27,35,38,39,44,47,51,52,56,82,90,91,100,104,111,121]	17
Decision tree	[10,20,33,38-41,58,72,73,81,97,98,111,117,123,126]	17
Naive bayes	[10,25,33,36,39,43,47,52,56,58,69,72,74,88]	14
Multilayer perceptron	[39,44,86,92,95,99,109,113,117,126]	10
Long short-term memory	[14,29,34,71,89,94,116,122,124]	9
Neural network	[27,32,72,88,97,105,108,125]	8
XGBoost	[7,94,98,105,108]	5
Support vector regressor	[45,49,91,109]	4
Bagged trees	[11,56,62,81]	4
CNN-LSTM^a^	[9,17,34,76]	4
AdaBoost	[9,17,58,104]	4
Linear discriminant analysis	[47,52,81,126]	4
Gaussian process regression	[69,83,109]	3
Lasso	[60,91,123]	3
Discriminant analysis	[40,41,43]	3
Gradient boosted tree	[14,34,121]	3
Light gradient boosted machine	[55,111]	2
Principal component analysis	[13,70]	2
Inception time	[19,65]	2
Gaussian mixture model	[24,28]	2
Boosted decision tree	[92,115]	2
Hidden Markov model	[28,45]	2
Denoising autoencoder	[9,17]	2
K-means	[113,118]	2
Deep neural network	[29,35]	2
Fully convolutional network	[65,107]	2
Residual neural network	[94,107]	2
Tree ensemble	[33,43]	2
Extra trees	[111]	1
Gradient boosting	[95]	1
Seq2Seq	[50]	1
Gaussian process	[117]	1
Bayesian regression	[91]	1
Ridge regression	[91]	1
ASRF^b^	[50]	1
Boosted ensemble decision trees	[112]	1
NN-DTW^c^	[82]	1
Recurrent neural network	[116]	1
Cluster tree	[52]	1
Generative kernel	[52]	1
Gated recurrent unit	[64]	1
t-SNE^d^	[106]	1
MLSTM-FCN^e^	[107]	1
MALSTM-FCN^f^	[107]	1
LRCF^g^	[84]	1
Nonnegative matrix factorization	[70]	1
Multidimensional scaling	[70]	1
Transformer	[29]	1
Baum-Welch	[28]	1
Random forest classifier	[119]	1
Logistic model trees	[25]	1
J48^h^	[25]	1
Neural network clustering	[22]	1
Gaussian naive bayes	[73]	1
Feedforward neural network	[45]	1
Multilayer Gaussian process	[37]	1
Radial basis function network	[66]	1
M5 prime algorithm	[44]	1
Blending ensemble learning	[120]	1
Generative adversarial network	[23]	1
Adaptive mixtures of local experts	[92]	1
Bayesian decision support	[92]	1
Sparse component analysis	[118]	1
Extreme learning machine	[79]	1
Naive bayes classifier	[68]	1
Structural equation modeling	[56]	1
Bayesian ridge regression	[109]	1
Gradient boosting algorithm	[73]	1
CRNN^i^	[102]	1

^a^CNN-LSTM: convolutional neural network followed by a long short-term memory layer.

^b^ASRF: action segment refinement framework.

^c^NN-DTW: neural network with dynamic time warping.

^d^t-SNE: t-distributed stochastic neighbor embedding.

^e^MLSTM-FCN: multidimensional long short-term memory followed by a fully convolutional network.

^f^MALSTM-FCN: multidimensional attention long short-term memory followed by a fully convolutional network.

^g^LRCF: local rank convolutional framework.

^h^J48: Java implementation of the C4.5 algorithm for decision trees.

^i^CRNN: convolutional recurrent neural network.

A significant majority of the models used, 232 (71.1%) in total, were supervised ML models, showcasing their widespread adoption in IMU data analysis. In contrast, unsupervised ML models were less commonly used, with only 15 (4.6%) papers reporting their application. Among these ML models, 5 methods stood out in terms of frequency, support vector machine was the most prevalent, then random forest, k-nearest neighbors, and decision trees followed and all had an average publication year of 2020. Only logistic regression had a slightly earlier average publication year of 2019.

These average publication years suggest that support vector machine, random forest, k-nearest neighbors, and decision trees, even if they are considered as old models, have been predominantly used in more recent studies. In contrast, the slightly earlier average year for logistic regression may reflect a decrease of interest on this model.

In the realm of DL, 72 (22%) models were supervised. Again, unsupervised DL models were considerably less common, with only 7 (2.1%) models used. The top 5 DL methods identified were convolutional neural networks (CNN), leading with an average publication year of 2020, multilayer perceptron (MLP) averaging at 2019, long short-term memory (LSTM) networks, with an average publication year of 2021, neural networks (NN), also averaging 2019, and CNN-LSTM hybrid models averaging at 2020.

These average publication years suggest that LSTM networks have seen increased adoption in the most recent studies (2021) over other models, likely due to their strength in handling sequential and time-series data inherent in IMU analyses. The consistent average years for CNN and CNN-LSTM models (2020) indicate sustained interest and applicability in feature extraction and sequence modeling. In contrast, the slightly earlier average years for MLP and general NNs (2019) may reflect earlier exploration phases or a shift toward more specialized architectures in recent research.

### Dataset Size

**Figure 3:**
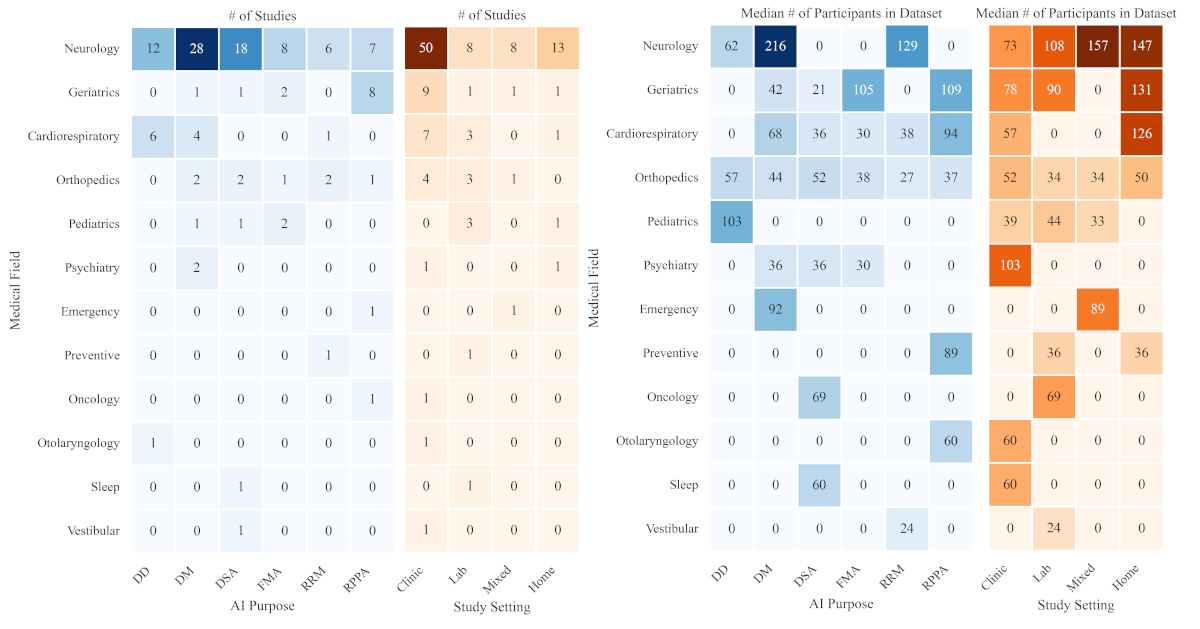
The left heatmap quantifies the volume of studies, while the right heatmap displays the median number of participants within datasets employed across these studies. The blue sides illustrate the diversity of machine learning applications—Disease Detection (DD), Disease Monitoring (DM), Disease Severity Assessment (DSA), Functional and Mobility Assessment (FMA), Rehabilitation and Recovery Monitoring (RRM), and Risk Prediction and Preventive Analysis (RPPA)—across various medical specialties. The orange sides visualize the data collection settings for ML training datasets, encompassing clinical, laboratory, home, and hybrid environments. The median number of participants was used to reduce the impact of the outliers with very big datasets.

The median dataset sizes varied significantly across different medical fields. The field of general and preventive medicine used the smallest median dataset size at 24, followed by pediatric medicine with 36 (IQR 32.5-36.5). Musculoskeletal and orthopedic care, neurology, and sleep medicine had median sizes of 38.5 (IQR 29.8-59.5), 45 (IQR 30-78.5), and 69, respectively. Vestibular medicine and sleep medicine both showed a median dataset size of 60. Larger median dataset sizes were observed in emergency medicine at 89, geriatric medicine at 91.5 (IQR 66.5-147.75), psychiatry at 91.5 (IQR 74.3-108.8), and pulmonary and cardiology medicine at 93.5 (IQR 42.5-130). The largest median dataset size was noted in otolaryngology, with 103 participants (Figure 3).

When examining the dataset sizes based on the purpose of ML application, varying trends were observed. Rehabilitation and recovery monitoring had the smallest median dataset size at 31.5 (IQR 24-42.3). This was followed by functional and mobility assessment at 38 (IQR 32-53), disease severity assessment at 49 (IQR 30-80.5), disease monitoring at 49.5 (IQR 29.3-103) and disease detection at 58 (IQR 44-86.5). The largest median dataset size, at 74 (IQR 52-109.5), was for risk prediction and preventive analysis.

The study settings also influenced the median dataset sizes. In clinic and real-life mixed settings, the median dataset size was 35 (IQR 30-69.5). Laboratory settings showed a similar median size of 35.5 (IQR 29.75-81.75). The clinic setting alone had a median size of 54.5 (IQR 38.25-83). Finally, the real-life setting presented the largest median dataset size of 60 (IQR 24-147).

Overall, the median dataset size across all studies was 50.5 (IQR 33.25-87.25), indicating a moderate scale of data used in training AI algorithms in these diverse settings and purposes." Under discussion section it is also 50.5 (IQR 33.25-87.25).

## Discussion

### Principal Findings

One of the most significant findings is the overall median dataset size of 50.5 (IQR 54) participants, and it raises significant concerns about the practical effectiveness of ML and, more so, DL methodologies in this context. These algorithms perform better with large datasets as they not only train more effectively but also ensure the generalizability of their outcomes across diverse populations and conditions [127]. The relatively small size of datasets in current research could limit the robustness and generalizability of ML and DL technologies, rather than their accuracy. Furthermore, even though there is no exact definition of what constitutes a “small” or “large” dataset, it is understood that the number must be large enough to represent the population adequately in order to generalize effectively [4], highlighting the need for a sufficiently large dataset.

Given the importance of participant variability for achieving good generalization, these findings about dataset size, or dataset variability, also highlight the importance of differentiating dataset length and dataset variability in the context of IMU data use of ML in health care. Dataset length refers to the acquisition of a broad number of data points from a participant (eg, 2 participants and 1000 datapoints per participant), which could potentially lead to overfitting due to the emphasis on big multidimensional arrays of features such as temporal patterns, spatial movements, or biometric signals. This focus might inadvertently prioritize the complexity of the dataset over its generalizability, suggesting that it could be less beneficial, and possibly even detrimental, compared to dataset variability which focuses on increasing the dataset by adding more participants and not more data per participant (eg, 50 participants and 40 datapoints per participant). Therefore, with a balance between dataset length and dataset variability, researchers could better leverage ML and DL methodologies to their full potential in this field.

The challenges highlighted above underline the need for combined efforts to expand the scope and scale of data collection in future studies. To assemble larger and more diverse datasets, researchers should consider multi-institutional collaborations or using new technologies for data collection and sharing. Furthermore, even with successful collection of wearable IMU data or data from other wearable devices, a significant obstacle remains in integrating this data into hospital systems or electronic health records [128]. The current lack of infrastructure and protocols for efficiently transferring and storing wearable device data within medical records systems not only impedes its immediate use in clinical decision making but also makes it challenging to gather data for future research and build large datasets for long-term patient monitoring and analysis. There’s a critical need for developing standardized protocols and systems capable of handling the varied data types produced by wearable technologies to ensure this valuable information is integrated into patient care workflows and health records management seamlessly. Finally, advancements in ML and DL techniques that are adept at working with smaller datasets, or that improve data augmentation practices, could play a crucial role in navigating these challenges. Such innovations could provide a pathway to maximize the use of wearable technologies in improving patient outcomes and propelling medical research forward, despite the current limitations posed by dataset sizes and integration issues.

Another finding indicates a substantial reliance on supervised machine learning methods, observed in 71.1% of the studies, which may also be attributed to the limited number of publicly available labeled datasets in this area. This highlights the reliance on labeled data for model training and validation. However, the labor-intensive process of data labeling, especially with complex medical datasets, poses significant challenges [129]. In this context, unsupervised learning methods are a compelling alternative, offering the advantage of discovering hidden patterns and correlations in data without the need for prelabeled instances. The application of unsupervised learning techniques can mitigate the bottleneck of data labeling, enabling researchers to leverage larger datasets more efficiently. This approach would not only facilitate a deeper understanding of the underlying data structures but also aid in identifying novel biomarkers and disease subtypes.

On the other hand, DL contains enormous potential for analyzing IMU medical data, particularly when fueled by larger datasets. DL’s capacity to model complex, nonlinear relationships, and its prowess in feature extraction from high-dimensional data make it exceptionally suited for IMU data analysis. The challenge of assembling substantial, well-annotated datasets in the medical domain, however, limits the full exploitation of deep learning’s capabilities. The availability of larger datasets, potentially facilitated by unsupervised learning and other innovative data gathering and processing techniques, would not only address this limitation but also propel forward the development of more sophisticated and accurate DL models. By enabling access to more extensive and diverse data, the research community could accelerate the advancement of deep learning methodologies, leading to breakthroughs in predictive accuracy, diagnostic reliability, and ultimately, patient outcomes.

Another interesting finding is that, on average, each study uses 2.67 ML or DL techniques. This statistic highlights the field’s ongoing experimental nature and its search for direction, as researchers actively explore a variety of algorithms and model architectures to identify the most effective methods for processing IMU data in medical applications. In addition, we found that for classification tasks the metrics used to evaluate these methods vary widely among studies. Some use accuracy, others use *F*_1_-score, and some rely on the area under the curve. This diversity in metrics makes it difficult to compare results directly and to ascertain which ML/DL models are most effective for medical research involving IMU data. The absence of standardized evaluation metrics is a significant barrier to effectively comparing and synthesizing results from various studies, thereby underlining the importance of establishing reproducibility and enabling scalable research methods.

To address these challenges, it is advised that the research community works together to develop standardized evaluation metrics. This collaborative effort could be enhanced by actively including discussions and presentations on these topics in existing conferences and congresses. By integrating these studies into broader scientific gatherings, it would promote consensus-building among researchers and clinicians, fostering the exchange of insights and best practices. Ultimately, the creation of baseline results and standardized evaluation metrics would streamline research efforts and markedly improve the reliability, generalizability, and clinical applicability of the findings.

The predominance of machine learning studies analyzing IMU data within controlled environments such as clinics and laboratories, which represents 77% of the research reviewed, highlights a critical focus on obtaining high-quality, precise data for specific disease studies. While beneficial for eliminating external variables that might affect the data’s integrity, this approach contrasts with the unpredictable nature of daily life where wearable IMUs are most impactful. The real value of wearable IMUs lies in their potential to continuously monitor health conditions in natural settings, capturing nuanced data on disease manifestation and progression. However, the transition from controlled environments to real-life application faces significant challenges, primarily due to technological limitations such as inadequate battery life and insufficient data storage capacity in wearable devices. For example, the UK Biobank, which holds the largest dataset of accelerometer data from patients, used the Axivity AX3 (Axivity) to collect data at high frequency but was only able to record up to 7 days of data before the battery was depleted or the memory was full, and only recording accelerometer data and no gyroscope. This limitation restricts the potential for capturing valuable longer-term trends, such as detecting subtle changes in activity patterns or sleep disturbances that might occur over a longer period, which could be critical for early diagnosis of conditions like Parkinson disease or monitoring gradual recovery after surgery. These goes alongside difficulties ensuring patient compliance outside of clinical settings, where frequent charging and maintenance of the devices may disrupt consistent monitoring.

Addressing these challenges requires advancements in wearable technology to enhance device usability and reliability in everyday settings, alongside strategies to improve patient engagement and adherence to usage protocols. Enhancing battery life and expanding storage without compromising device comfort, coupled with the development of intuitive interfaces, could significantly boost patient compliance and data collection quality. Furthermore, leveraging machine learning algorithms to manage data and power more efficiently, and implementing patient education and motivation strategies, could bridge the gap between controlled studies and the dynamic context of real-world scenarios. By overcoming these technological and logistical problems, researchers can unlock deeper insights into health conditions through wearable IMUs, paving the way for more personalized and effective health care solutions that reflect the complexity of real-life patient experiences.

Finally, the fact that 64.7% of the studies focused on neurological conditions underscores a notable trend in the scientific community, particularly regarding the use of IMUs for analyzing movement disorders related to Parkinson disease. This focus is likely because neurological disorders exhibit distinct movement disorders, which can be readily detected and analyzed by Inertial IMUs and ML algorithms, leveraging their capacity to identify clear patterns in movement data. However, this concentration on neurology suggests an unintentional narrowness in application, potentially overlooking the vast possibilities in other medical fields.

Expanding the application of IMUs and ML/DL to include other medical areas, like musculoskeletal care, represents a promising avenue to harness the full potential of these technologies. To rectify this imbalance and encourage a broader application spectrum, targeted funding initiatives and interdisciplinary research collaborations could be established. These efforts would incentivize exploration into how IMUs and ML/DL can revolutionize diagnostics, treatment planning, and outcome monitoring. By diversifying research directions, the medical community can ensure a more equitable distribution of technological advancements across various domains, maximizing the impact of these cutting-edge tools in improving patient care and treatment outcomes.

### Limitations

Despite its strengths, our systematic review has some limitations. While our strict inclusion criteria ensured high-quality research, it may have excluded some relevant studies. Furthermore, using an inclusion criterion of “study targets a clinical condition” will remove useful studies like sleep analysis or human activity recognition, but that is not the scope of this review. In addition, the variability in study quality is a common challenge in systematic reviews, and we prioritized datasets with a minimum number of sample size to increase confidence in our conclusions.

### Conclusions

In conclusion, this systematic review has provided valuable insights into the current state of ML and DL applications on IMU data in the medical field. The key findings highlight several critical aspects: the relatively small median dataset size of 50.5 participants, the dominance of supervised ML methods, the exploratory nature of method selection in research, the preference for clinical settings over real-life data collection, and the focus on neurological problems. These findings suggest that while the integration of ML and DL in medical research is progressing, there is still considerable room for growth and optimization. The challenges identified, such as limited dataset sizes and the need for more real-world data, must be addressed to fully realize the potential of these technologies in health care.

## Appendix

Multimedia Appendix 1 PRISMA (Preferred Reporting Items for Systematic Revues and Meta-Analyses) main and abstract checklists.

Multimedia Appendix 2 Description for all Artificial Intelligence Models reviewed.

## Abbreviations

AI: artificial intelligence
CNN: convolutional neural network
DL: deep learning
IMU: inertial measurement unit
LSTM: long short-term memory
ML: machine learning
MLP: multilayer perceptron
NN: neural network
PRISMA: Preferred Reporting Items for Systematic Revues and Meta-Analyses
