# Supplementary material for: The Use of Artificial Intelligence and Wearable Inertial Measurement Units in Medicine: Systematic Review
Source: JMIR Mhealth Uhealth. 2025 Jan 29;13:e60521. doi: 10.2196/60521 (PMC11822330; doi:10.2196/60521)
Supplement: Multimedia Appendix 2 [file mhealth_v13i1e60521_app2.docx]

# APPENDIX

## Machine Learning

### Supervised Learning

- AdaBoost: Combines multiple weak classifiers to form a strong classifier.
- BEDT (Boosted Ensemble Decision Trees): Enhances performance by combining multiple decision trees.
- BDT (Boosted Decision Tree): Uses boosting to improve decision tree accuracy.
- BEL (Blending Ensemble Learning): Merges predictions from different models for better performance.
- BR (Bayesian Regression): Incorporates prior knowledge into regression analysis.
- BRR (Bayesian Ridge Regression): Regularizes linear regression with a Bayesian approach.
- BT (Bagged Trees): Reduces variance by averaging multiple decision trees.
- DA (Discriminant Analysis): Classifies by finding a linear combination of features.
- DT (Decision Tree): Splits data into branches based on feature values.
- ELM (Extreme Learning Machine): Fast learning algorithm for single-layer feedforward neural networks.
- ET (Extra Trees): Uses random splits in tree nodes for diverse models.
- GB (Gradient Boosting): Builds models sequentially to minimize prediction errors.
- GBA (Gradient Boosting Algorithm): Iteratively refines predictions by correcting errors.
- GBT (Gradient Boosted Tree): Combines decision trees with gradient boosting.
- GTBM (Gradient Tree Boosted Machine): Enhances trees using gradient boosting techniques.
- GNB (Gaussian Naive Bayes): Assumes normal distribution for features.
- GP (Gaussian Process): Non-parametric approach for regression and classification.
- GPR (Gaussian Process Regression): Uses Gaussian processes for regression tasks.
- J48: Java implementation of the C4.5 algorithm for decision trees.
- kNN (k-Nearest Neighbors): Classifies based on the majority label of nearest neighbors.
- Lasso (Lasso Regression): Performs linear regression with L1 regularization.
- Lasso LR (Lasso Logistic Regression): Applies Lasso regularization to logistic regression.
- LDA (Linear Discriminant Analysis): Projects data to maximize class separability.
- LGBM (Light Gradient Boosting Machine): Efficient gradient boosting for large datasets.
- LMT (Logistic Model Trees): Combines logistic regression and decision trees.
- LR (Logistic Regression): Predicts binary outcomes using a logistic function.
- M5P (M5' model trees): Constructs model trees for regression tasks.
- NB (Naive Bayes): Classifies using Bayes' theorem with strong independence assumptions.
- NBC (Naive Bayes Classifier): Simple probabilistic classifier based on Bayes' theorem.
- RF (Random Forest): Aggregates multiple decision trees to improve accuracy.
- RFC (Random Forest Classifier): Random Forest algorithm for classification tasks.
- RR (Ridge Regression): Regularizes linear regression with L2 penalty.
- SVM (Support Vector Machine): Finds the optimal hyperplane for classification.
- SVR (Support Vector Regression): Extension of SVM for regression problems.
- TE (Tree Ensemble): Combines multiple trees to improve predictive performance.
- XGB (XGBoost): Optimized gradient boosting library for superior performance.

### Unsupervised Learning

- BDS (Bayesian Decision Support): Uses Bayesian statistics for decision-making under uncertainty.
- BW (Baum-Welch): An algorithm to train Hidden Markov Models.
- CKA (Consensus K-Means Analysis): Combines multiple K-Means runs to improve clustering stability.
- GMM (Gaussian Mixture Model): A probabilistic model for representing normally distributed subpopulations.
- HMM (Hidden Markov Model): Models systems that are a Markov process with hidden states.
- KM (K-Means): A clustering algorithm that partitions data into K distinct clusters.
- MDS (Multidimensional Scaling): Reduces dimensionality while preserving pairwise distances.
- NMF (Non-negative Matrix Factorization): Factorizes a matrix into non-negative factors.
- PCA (Principal Component Analysis): Reduces dimensionality by transforming data into a new set of orthogonal components.
- SCA (Sparse Component Analysis): Identifies sparse components in the data.
- SEM (Structural Equation Modeling): Analyzes structural relationships between measured variables.
- t-SNE (t-distributed Stochastic Neighbor Embedding): Reduces dimensionality for visualization, preserving local structure.

## Deep Learning

### Supervised Learning

- AMM (Adaptive Mixtures of Local Experts): Combines multiple models to make predictions.
- ASRF (Action Segment Refinement Framework): Enhances action detection in sequences.
- CNN (Convolutional Neural Network): Specialized for processing grid-like data such as images.
- CNN-LSTM (Combination of CNN and LSTM): Integrates CNN and LSTM for spatiotemporal data.
- CRNN (Convolutional Recurrent Neural Network): Merges CNNs and RNNs for sequential data processing.
- DNN (Deep Neural Network): A neural network with multiple hidden layers.
- FCN (Fully Convolutional Network): Utilizes convolutional layers for end-to-end tasks.
- FFNN (Feedforward Neural Network): Basic neural network where connections do not form cycles.
- GAN (Generative Adversarial Network): Consists of two networks competing to generate realistic data.
- GRU (Gated Recurrent Unit): A type of RNN that can capture long-term dependencies.
- Inception (A type of CNN for Time Series data): Efficient CNN architecture for complex models.
- LSTM (Long Short-Term Memory): An RNN variant for learning long-term dependencies.
- MALSTM-FCN (Multi-dimensional Attention LSTM followed by a Fully Convolutional Network): Combines attention mechanisms, LSTM, and FCN for complex data.
- MLGP (Multilayer Gaussian Process): Combines Gaussian processes for deep learning.
- MLP (Multilayer Perceptron): A simple feedforward neural network with multiple layers.
- MLSTM-FCN (Multi-dimensional LSTM followed by a Fully Convolutional Network): Blends LSTM and FCN for multidimensional data.
- NN (Neural Network): A basic model inspired by biological neural networks.
- RBFN (Radial Basis Function Network): Uses radial basis functions as activation functions.
- ResNet (Residual Neural Network): Utilizes skip connections to ease training of deep networks.
- RNN (Recurrent Neural Network): Designed for sequential data, with cycles in the network.
- Seq2Seq (Sequence to Sequence): Converts sequences from one domain to another.
- Transformer: Processes sequences in parallel using self-attention mechanisms.

### Unsupervised Learning

- CKA (Convolutional Kernel Analysis): A method for comparing neural network representations using kernel-based similarity.
- CT (Cluster Tree): A hierarchical clustering algorithm that organizes data into a tree structure.
- DAE (Denoising Autoencoder): A type of autoencoder trained to remove noise from input data.
- GK (Generative Kernel): A technique that combines kernel methods with generative models.
- LRCF (Local Rank Convolutional Framework): A framework that applies convolutional operations based on local rank information.
- NN-Clustering (Neural Network Based Clustering): Clustering method that utilizes neural network architectures.
- NN-DTW (Neural Network with Dynamic Time Warping): Combines neural networks with dynamic time warping for temporal sequence analysis.
- RRelief (Relief algorithm with modifications): An enhanced version of the Relief algorithm for feature selection.
